# Cannabidiol and Cannabigerol Exert Antimicrobial Activity without Compromising Skin Microbiota

**DOI:** 10.3390/ijms24032389

**Published:** 2023-01-25

**Authors:** Mariana Luz-Veiga, Manuela Amorim, Inês Pinto-Ribeiro, Ana L. S. Oliveira, Sara Silva, Lígia L. Pimentel, Luís M. Rodríguez-Alcalá, Raquel Madureira, Manuela Pintado, João Azevedo-Silva, João Fernandes

**Affiliations:** 1 Centro de Biotecnologia e Química Fina (CBQF)—Laboratório Associado, Escola Superior de Biotecnologia, Universidade Católica Portuguesa, Rua Diogo Botelho 1327, 4169-005 Porto, Portugal; 2 Amyris Bio Products Portugal, Unipessoal Lda, Rua Diogo Botelho 1327, 4169–005 Porto, Portugal

**Keywords:** cannabidiol, cannabigerol, antimicrobial activity, biofilm, cosmetic preservative, keratinocytes, skin microbiota

## Abstract

Cannabidiol (CBD) and cannabigerol (CBG) are two pharmacologically active phytocannabinoids of *Cannabis sativa* L. Their antimicrobial activity needs further elucidation, particularly for CBG, as reports on this cannabinoid are scarce. We investigated CBD and CBG’s antimicrobial potential, including their ability to inhibit the formation and cause the removal of biofilms. Our results demonstrate that both molecules present activity against planktonic bacteria and biofilms, with both cannabinoids removing mature biofilms at concentrations below the determined minimum inhibitory concentrations. We report for the first time minimum inhibitory and lethal concentrations for *Pseudomonas aeruginosa* and *Escherichia coli* (ranging from 400 to 3180 µM), as well as the ability of cannabinoids to inhibit *Staphylococci* adhesion to keratinocytes, with CBG demonstrating higher activity than CBD. The value of these molecules as preservative ingredients for cosmetics was also assayed, with CBG meeting the USP 51 challenge test criteria for antimicrobial effectiveness. Further, the exact formulation showed no negative impact on skin microbiota. Our results suggest that phytocannabinoids can be promising topical antimicrobial agents when searching for novel therapeutic candidates for different skin conditions. Additional research is needed to clarify phytocannabinoids’ mechanisms of action, aiming to develop practical applications in dermatological use.

## 1. Introduction

Cannabinoids are a group of substances that can bind to cannabinoid receptors (i.e., CB_1_ and CB_2_) and modulate the activity of the endocannabinoid system (ECS) [1]. These can be endogenous to the body (endocannabinoids), chemically synthesized, or isolated from the *Cannabis sativa* L. plant (phytocannabinoids) [1,2]. More than 100 different phytocannabinoids have been identified so far [3], with THC and cannabidiol (CBD) being the most abundant cannabinoids in the plant [4]. Other cannabinoids of the same origin include cannabigerol (CBG), cannabinol (CBN), cannabichromene (CBC), and cannabigerovarin (CBGV) [1], albeit most research has been mainly focused on CBD and THC.

Cannabidiol has been described as exerting a variety of beneficial pharmacological effects, including anti-inflammatory, antioxidant, and neuroprotective properties [5,6,7]. It is currently in the advanced stages of clinical testing for acne treatment and has also been approved for the treatment of severe seizures in epilepsy [8,9,10]. Cannabidiol’s antimicrobial activity also stands out—specifically, its activity against a wide range of Gram-positive bacteria, including a variety of drug-resistant strains such as methicillin-resistant *Staphylococcus aureus* (MRSA), multidrug-resistant *Streptococcus pneumoniae*, *Enterococcus faecalis*, and the anaerobic bacteria *Clostridioides* (previously *Clostridium*) *difficile* and *Cutibacterium* (formerly *Propionibacterium*) *acnes* [11,12,13,14,15]. This effect is believed to be associated with a disruption of the bacterial membrane [11], but further studies are still required to fully elucidate this question.

Cannabigerol acts as the precursor molecule for the most abundant phytocannabinoids, including CBD and THC. It has attracted some interest, with recent reports demonstrating it activates alpha(2)-adrenoceptors, blocks serotonin 1A (5-HT_1A_) and CB_1_ receptors, and binds to CB_2_ receptors, potentially having neuroprotective effects [16,17]. Similarly to CBD, CBG has also been studied for its antibacterial properties, with studies showing activity against methicillin-resistant *S. aureus* (MRSA) [18] and planktonic growth of *Streptococcus mutans* [19]. Furthermore, CBG is also capable of interfering with the quorum sensing-mediated processes of *Vibrio harveyi*, resulting in the prevention of biofilm formation [20].

Cannabinoids’ antimicrobial effect upon key pathogens of the skin (e.g., *Staphylococci*, *Streptococci* and *Cutibacterium* genus) is of note, as certain inflammatory skin conditions are triggered or at higher risk of infection by *S. aureus* and *S. pyogenes* [21,22]. The association between streptococcal infection and guttate psoriasis has been well established, and disease exacerbation has been linked to skin colonization by *S. aureus* and *Candida albicans* [21,23]. Another example is atopic dermatitis, whose severity has been correlated to toxin production by *S. aureus* strains, and their superantigens also have an aggravating role [24].

Considering the current knowledge, we aimed to elucidate CBD and CBG interaction and potential antimicrobial activity upon selected microorganisms, namely on human-skin-specific microorganisms commonly associated with inflammatory skin conditions. Furthermore, the impact of these compounds on the establishment of pathogenic biofilms and their capacity to inhibit keratinocytes’ infection were also a target of this research effort. Finally, considering a potential topical use for skin conditions, dermocosmetic formulations with CBD and CBG were prepared and studied for antimicrobial preservation efficacy and for their impact upon skin microbiota and skin homeostasis.

## 2. Results

### 2.1. CBD and CBG Purity and Chemical Analysis

**Figure 1:**
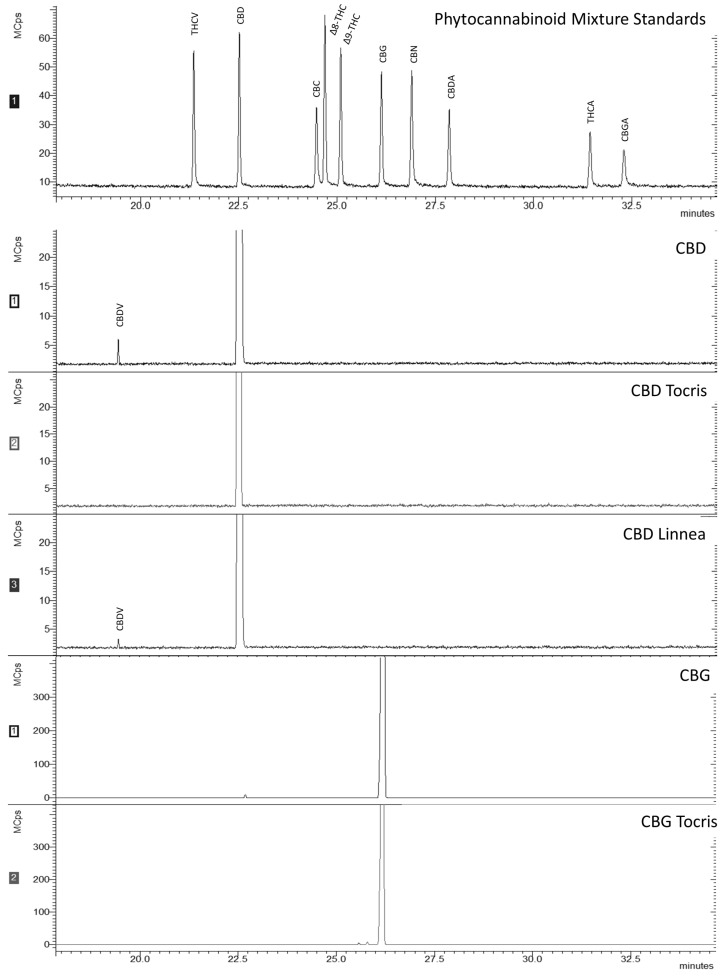
Chromatograms of CBD and CBG samples vs. Phytocannabinoids Standards. All peaks are annotated as follows: THCV: tetrahydrocannabivarin, CBD: cannabidiol, CBC: cannabichromene, CBDV: cannabidivarin, Δ8-THC: Δ8-tetrahydrocannabinol, Δ9-THC: Δ9-tetrahydrocannabinol, CBG: cannabigerol, CBN: cannabinol, CBDA: cannabidiolic acid, THCA: tetrahydrocannabinolic acid, and CBGA: cannabigerolic acid.

Concerning the purity of the phytocannabinoids used in this work, it is possible to conclude that all samples have an average purity of 99% (*p* > 0.05; Table A4). Moreover, in the chromatographic results (Figure 1) for the samples CBD and CBD Linnea, another compound was detected. This compound, although not present in the phytocannabinoid mixture standards, was identified as cannabidivarin (CBDV) by comparing the spectrum obtained with that described in SpectraBaseTM. In both mass spectra, the following fragments were found with m/z: 73 (characteristic of TMS derivatives), 430 (molecular ion), as well as 273 and 362. As concerns the chromatographic results of CBG samples, both samples showed a purity of 99% (*p* > 0.05), as expected. Nevertheless, in the CBG Tocris sample, two peaks were detected at 25.6 and 25.8 min next to that of CBG. These compounds were not identified as no match was found in the consulted databases. However, these might be CBG analogues.

### 2.2. CBG and CBD Hinder Microbial Growth

The phytocannabinoids demonstrated an antimicrobial effect for all tested microorganisms, with MICs registered for all bacteria and fungi. Results are presented in Table 1. For most cannabinoids assayed, MIC values ranged from 10 to 100 µM for Gram-positive bacteria, with lethal concentrations ranging from 50 to 100 µM, while for Gram-negative bacteria, MIC values ranged between 400 and 1000 µM, with MLCs ranging from 3180 to 5000 µM.

Overall, the MIC values obtained for the Gram-positive were at least five times higher than those obtained using antibiotics as a control. The only exception was *C. acnes*, a highly fastidious type of bacteria, which demonstrated a much higher MIC (ranging from 300 to 1000 µM) and MLC (3180 to 5000 µM) for both CBG and CBD. These results were 300 times higher than MIC values attained for vancomycin and ciprofloxacin.

Regarding the Gram-negative bacteria tested, the MIC and MLC values obtained were at least >10-fold higher than those observed for Gram-positive bacteria. Moreover, at the MIC level, results varied for the different cannabinoids tested, whereas MLC concentrations were consistent for both *E. coli* and *P. aeruginosa*, standing between 3180 and 5000 µM for all cannabinoids. For example, for *P. aeruginosa,* MICs were registered for CBG and CBG Tocris at 400 µM, although for *E. coli*, values ranged from 500 µM for CBG to 1000 µM for CBG Tocris. Here, the concentrations attained were between 100 and 300 times higher than those obtained for the tested antibiotics. Finally, it was also possible to establish MICs and MLCs for all compounds regarding the yeast tested, *C. albicans.* CBG, CBD, and CBD Linnea had the same MIC (200 µM), while for CBD Tocris, it was 250 µM, and for CBG Tocris, it was determined as 400 µM, with MLCs standing between 300 and 500 µM for all cannabinoids. Regarding the solvent control, it did not lead to any inhibitions for any of the microorganisms tested.

### 2.3. CBG and CBD Inhibit Biofilm formation

**Figure 2:**
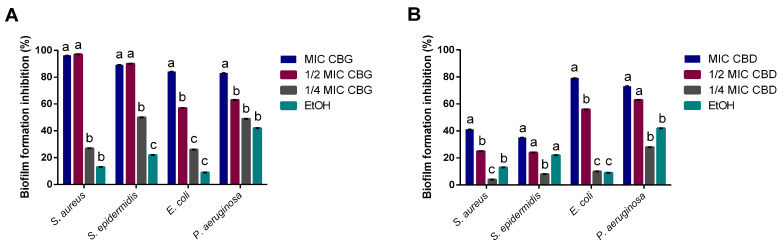
Biofilm formation inhibition percentage for CBG (**A**) and CBD (**B**) over *Staphylococci*, *E. coli*, and *P. aeruginosa*. Ethanol was used as a solvent control. Data are represented as mean  ±  SD for 2 independent assays encompassing 4 replicates. Letters mark statistically significant differences (*p* < 0.05) for each microorganism.

CBG fully inhibited the establishment of *Staphylococci* biofilm at the highest concentrations tested, namely MIC and ½ MIC (96 and 97% of inhibition, respectively, as seen in Figure 2). Further, CBG reduced *E. coli* and *P. aeruginosa* biofilm formation in a dose-dependent manner, where the use of the MIC led to approximately an 80–85% reduction, while using only ½ MIC led to a 57–63% reduction. Regarding CBD, it showed a lower impact on Gram-positive than on Gram-negative bacteria. For the *Staphylococci*, CBD MIC led to a 41% inhibition, less than half of the CBG effect. Additionally, the 25% inhibition for ½ MIC was not significantly different from the inhibition attained with ethanol. However, it was able to hamper *E. coli*’s biofilm formation at 79% and *P. aeruginosa* at 73% at MIC levels. The solvent control tested, ethanol, did not showcase a significant effect on the *Staphylococci*, with inhibitions ranging between 9% and 22%. However, it did lead to an inhibition of almost 40% for *P. aeruginosa*. Nevertheless, the inhibitions observed when using ethanol are significantly lower (*p* < 0.05) than the ones observed for CBG and CBD at MIC.

### 2.4. CBG and CBD Disrupt Mature Biofilms

**Figure 3:**
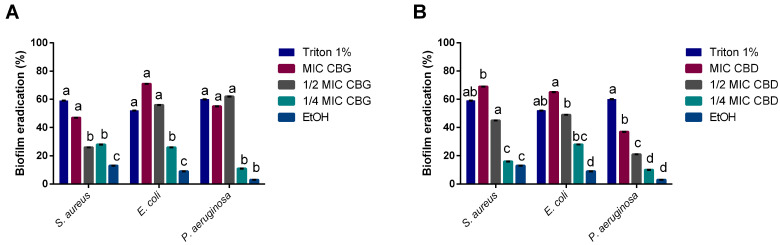
Biofilm eradication percentage caused by CBG (**A**) and CBD (**B**) over *S. aureus*, *E. coli*, and *P. aeruginosa.* Ethanol was used as a solvent control. Triton at 1% (*v*/*v*) was used as an eradication control. Data are represented as mean  ±  SD for 2 independent experiment encompassing 4 replicates. Letters mark statistically significant differences (*p* < 0.05) for each individual microorganism.

For all bacteria tested, the biofilm eradication values ranged from 52% to 71%. The use of the MICs and ½ MICs of both cannabinoids showed a statistically similar effect to Triton X-100, a detergent commonly used to dissociate mature biofilms (Figure 3). CBG’s effect upon *S. aureus* was the exception, as only the MIC had a similar effect to Triton (*p* > 0.05). Moreover, CBD’s MIC induced a significantly higher biofilm eradication percentage than CBG against *S. aureus* (69% and 47%, respectively). Overall, the solvent control (ethanol) did not yield eradication values above 13%, which was statistically significantly lower than the eradication caused by effective phytocannabinoid concentrations (i.e., MICs and ½ MICs). Thus, when considering the biofilm eradication assay, unlike what occurred in the biofilm inhibition assay, the solvent control did not interfere significantly with the bacteria. This result could be due to the biofilm already being strongly established and less susceptible to outside disruptions. Additionally, *S. epidermidis* results were not considered as the growth control was not significant.

### 2.5. CBD and CBG Impaired S. aureus Adhesion to Keratinocytes

**Figure 4:**
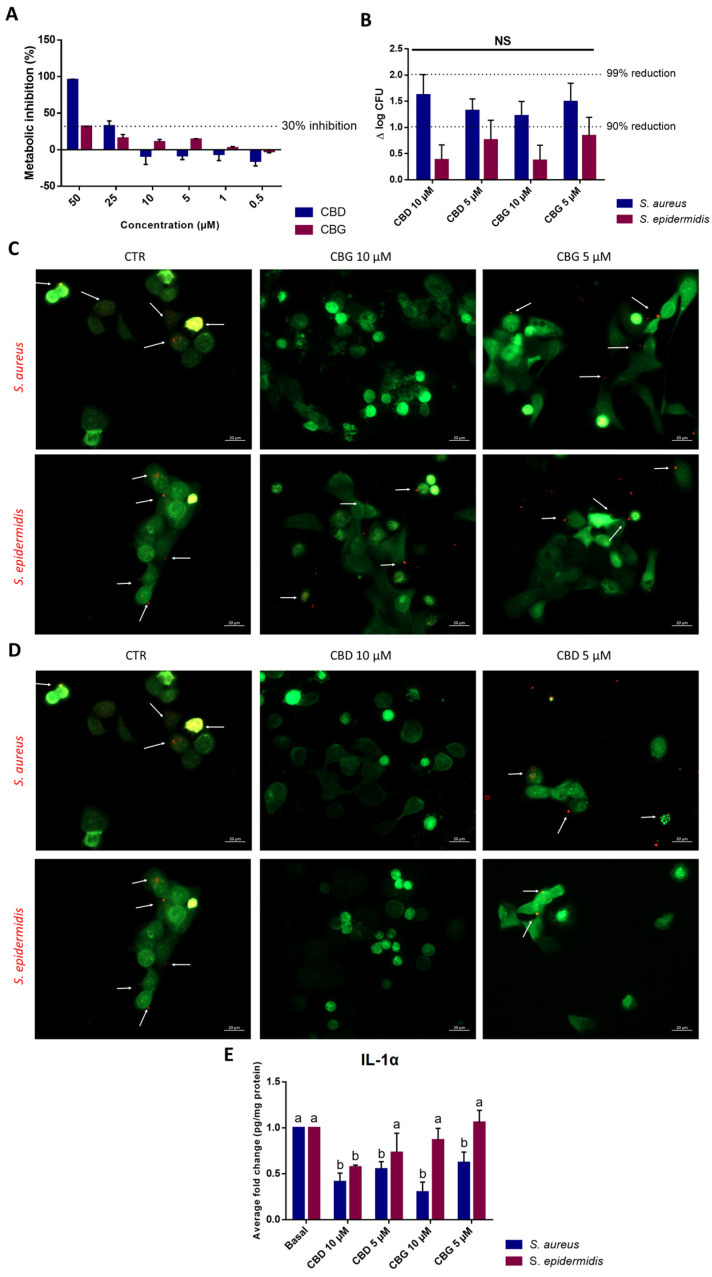
(**A**). Results for the biocompatibility assay with cannabinoids and HaCaT. Data represented as mean  ±  SD for *n* = 3 independent experiments. (**B**). Results for the adhesion assay with *Staphylococci*. A log cycle represents a 90% reduction, while 2 log cycles represent a 99% reduction in bacteria. Data are delta mean log CFU ± SD for *n* = 4 biologically independent samples. No significant differences between samples (*p* > 0.05) were found. (**C**,**D**). Microscopy results for the adhesion assay with *Staphylococci* with CBG (**C**) and CBD (**D**). *S. aureus* and *S. epidermidis* were labeled with Cell Tracker red, whereas HaCaT were marked with Cell Tracker green. Arrows indicate bacteria that adhered to cells. Images were taken at a total magnification of ×400. (**E**). Evaluation of cannabinoids’ effect on inhibiting inflammatory cytokine IL−1α in HaCaT cells was performed by ELISA. Results are presented as fold change to the basal condition (cells with bacteria), with data normalized to cytokine concentration per mg of protein. Letters mark statistically significant differences (*p* < 0.05) for each individual microorganism. Each value represented mean ± SD of 4 replicas for 2 independent experiments.

To test cannabinoids’ impact upon *Staphylococci* adhesion to keratinocytes, 10 and 5 µM were the selected concentrations, as these concentrations were not cytotoxic (Figure 4A; acc. ISO 10993-5 cytotoxic effect > 30% inhibition) [25]. As seen in Figure 4B, both compounds led to a higher than 90% reduction in *S. aureus* adhesion to HaCaT, equivalent to >1.0 log-cycle reduction in viable bacterial counts. However, no significant differences (*p* < 0.05) were observed between the two concentrations tested. The reduction noted for *S. epidermidis* was not sufficient to reach a log cycle. Albeit not significant, the depletion of both bacteria was visible on the fluorescence microscopy images using cell tracker probes (Figure 4C,D). As cannabinoids have been described as exerting anti-inflammatory activity, the conditioned medium of co-cultures was retrieved, and pro-inflammatory cytokines IL-1α, IL-6 and IL-8 were quantified, as these are commonly associated with skin inflammation. Cannabinoids led to a statistically significant reduction in IL-1α levels on the *S. aureus* supernatants. Regarding *S. epidermidis,* the detected IL-1α levels were not statistically different from the levels obtained for keratinocytes not treated with cannabinoids (basal condition) (Figure 4E). The values obtained for IL-6 and IL-8 were below the kit’s detection limit.

### 2.6. Evaluation of CBD and CBG as a Preservative in Cosmetic Formulations

**Figure 5:**
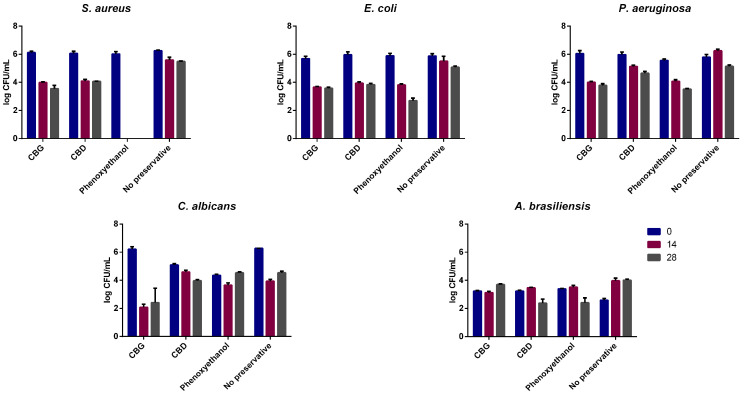
Results for the challenge test for the three bacteria and two fungi tested. These are described in log CFU/mL at 1, 14, and 28 days’ time points. Phenoxyethanol was used as a positive control. A formulation with no preservative was also tested. Data are *n* = 2 independent experiments and represented by mean log CFU ± SD.

The USP Chapter 51 Preservative Challenge Test is the most common method used to gauge preservative effectiveness, assessing the effect of preservatives in cosmetics, personal care products, and drug products. The microorganisms tested are known as contaminant strains [26]. Results are shown in Figure 5.

As seen in Table 2, CBG fulfilled the challenge test pass criteria: ≥2.0 log reduction from the initial calculated count at 14 days, and no increase from the 14 days’ count at 28 days for all the three bacteria, while no increase (≥0.5 log) was seen at 14 and 28 days for *C. albicans or A. brasiliensis.* Additionally, CBG impacted *C. albicans* growth, with a two-log-cycle reduction occurring after 14 days. Regarding CBD, it did not pass the criteria for *P. aeruginosa*, as the reduction on the number of viable bacteria was not ≥2.0 log cycles on day 14. However, it met all the other pass criteria for the remaining bacteria, yeasts, and molds.

Phenoxyethanol, a preservative used in many cosmetics and personal care products, was used as a positive control, meeting the criteria for all microorganisms except *P. aeruginosa*. It demonstrated a particularly strong effect on *S. aureus*, in which no growth was verified at any of the timepoints assayed (14 and 28 days). Finally, the blank formulation met the criteria for *C. albicans*, while it failed for the remaining microorganisms. This result may be due to the lack of nutrients present in the formulation, which might lead to microbial death over time.

### 2.7. CBG and CBD Have No Significant Impact on Skin Microbiota

**Figure 6:**
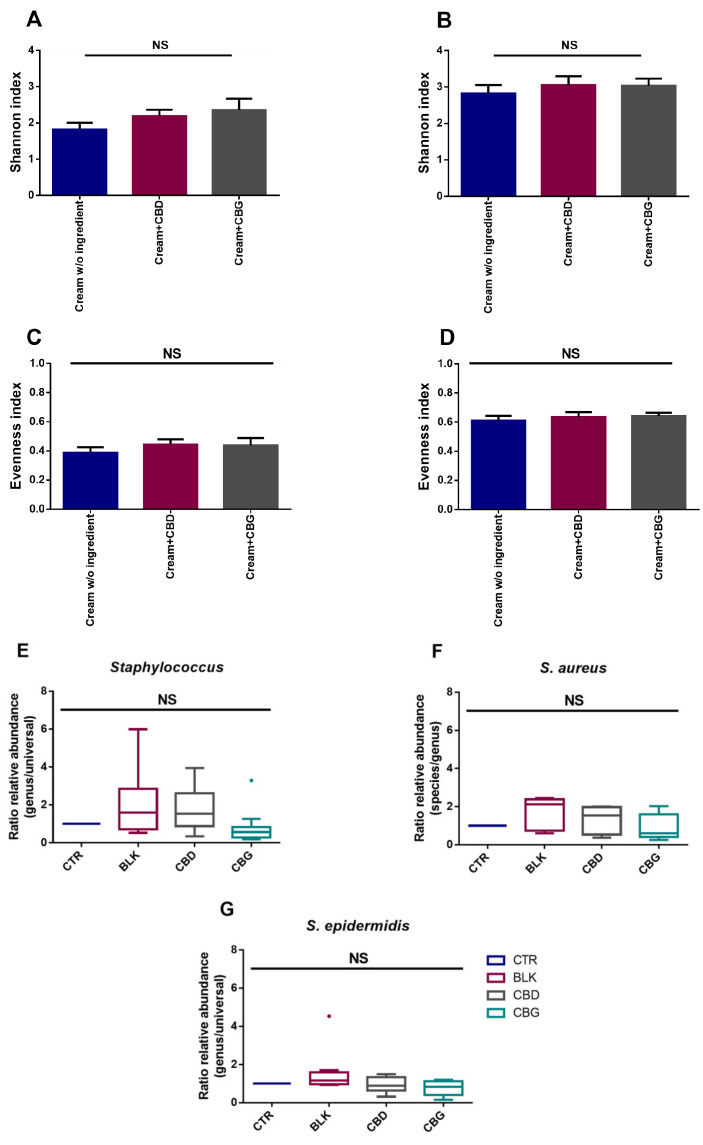
Alpha-diversity metric Shannon index demonstrated that the bacterial (**A**) and fungal (**B**) composition of samples was similar among all groups (*p* > 0.05). Alpha-diversity metric Evenness index demonstrated that the bacterial (**C**) and fungal (**D**) composition of samples was similar among all groups (*p* > 0.05). qPCRs results are presented as the ratio of relative abundance between genus and total bacteria (**E**) or between species and genus (**F**,**G**). No significant differences were found between samples (*p* > 0.05). The number of donors was 14; only 4 had detectable amounts of *S. aureus*.

The impact of CBD and CBG on the skin microbiota of healthy female volunteers was assessed. The alpha diversity was quantified through the Shannon and Evenness indexes (Figure 6). The Evenness index considers the relative abundance of the species. The Shannon index is related to species diversity and links the number of species living in a habitat (richness) with their relative abundance (evenness). Both alpha-diversity metrics demonstrated that the microbial composition of samples was similar between all groups, and no statistically significant differences were observed regarding bacteria and fungi (*p* > 0.05). Overall, the microbial profile demonstrated a high relative abundance of all commonly found phyla of the skin, including Actinobacteria, Firmicutes, and Basidiomycota, with *Staphylococcus* and *Malassezia* being the most abundant genera found regarding the bacterial and fungal community, respectively. Thus, the NGS results demonstrated that neither CBD nor CBG had a negative impact on the skin microbiota of the healthy volunteers included in this study. As the NGS results did not allow studying the microbial profile at the species level, qPCR was performed for *Staphylococci* species. Concerning these results, as can be seen in Figure 6E–G, although a slight decrease in the ratio can be seen for CBG, no significant differences (*p* > 0.05) were found between the samples regarding the relative abundance of *Staphylococci*.

## 3. Discussion

Cannabinoids have been described as possessing antimicrobial effects, although the mechanisms of action are not yet fully disclosed [11]. While CBD’s potential as an antimicrobial has been extensively studied, there is a lack of studies characterizing the activity of CBG [27]. Here, we demonstrated CBG and CBD’s strong antimicrobial activity, showing their potential as preservative agents and their interaction with human skin microorganisms. To the best of our knowledge, this is the first report with a MIC and MLC determination for both CBG and CBD against *S. pyogenes*, *P. aeruginosa*, and *E. coli*, as well as confirmed biofilm inhibition for both *P. aeruginosa* and *E. coli*.

Regarding the MIC and MLC assays, our study stands in line with previous reports, where CBD showed a strong activity upon Gram-positive bacteria, albeit with higher concentrations for some of the bacteria tested [11]. CBD’s impact upon *S. aureus* has been described in concentrations ranging from 0.03 to 1.57 μM [11,28], while those presented here range from 10 to 75 µM. Additionally, it is interesting that the MICs and MBCs attained for the Gram-positive bacteria were not far superior to the values obtained for the antibiotics, including vancomycin, which is a last-resort antibiotic. Both MIC and MBC were determined for *C. acnes* with all tested cannabinoids, except CBD Tocris. Reports have demonstrated CBD’s antimicrobial effect, and clinical trials are ongoing for acne treatment by topical application (NCT03573518) [29,30]. Nevertheless, this is the first study reporting CBG’s interaction with this bacterium. Concerning the yeast tested, although Feldman et al. [31] demonstrated CBD’s ability to inhibit *C. albicans* biofilm formation and to disrupt mature biofilm through a multitarget course of action, the author reported neither MIC nor MLC for planktonic *C. albicans.* Moreover, no studies have shown CBG’s impact on yeast or fungi, with this being the first time reported.

MIC and MLC values for the same microorganism differed from cannabinoid to cannabinoid. This may be due to differences in the strains used, the methodology employed, or the cannabinoids themselves, as different origins/extraction and purification methods can considerably impact results. For instance, all cannabinoids tested revealed a strong antimicrobial activity, although the values demonstrated in the antimicrobial results differed from molecule to molecule, sometimes significantly. For example, MIC for *S. aureus* was 10 µM for CBD and CBD Linnea (both purified from hemp seeds), whereas for CBD Tocris (prepared by chemical synthesis), it was 7 times higher (75 µM). For CBG Tocris, also prepared by chemical synthesis, MIC was 10 µM, while for CBG, it was 2.5 times higher (25 µM, the concentration corresponding to the subsequent twofold dilution). Those differences are probably due to the different sources of CBDs and to the nature of their impurities. This indication is corroborated by the GC-MS results (Figure 1), as the spectra for CBD and CBD Linnea also had a peak pertaining to CBDV. CBDV is a propyl analogue of CBD, also found in *C. sativa* L. [32,33]. Nevertheless, it has been demonstrated that the differences in MIC (or MLC) values are recurrent in studies with cannabinoids, especially when different origins and purity levels, combinations with antibiotics, or cannabis extracts are tested [12].

Cannabinoids’ impact on bacterial biofilms was also studied, as the capacity to inhibit biofilm formation or eradicate mature biofilms is of clinical relevance [34]. CBD’s moderate ability to inhibit biofilm formation for *S. aureus* diverges from what has been found in previous reports [11], showing only a 40% of biomass reduction. This could be a result of the strain used (*S. aureus* ATCC 6538™ vs. *S. aureus* ATCC 25923™) or the concentrations tested, as the ones that had an effect were much higher than the MIC obtained for *S. aureus* in this study. However, it did lead to high inhibitions of biofilm formation for *E. coli* and *P. aeruginosa* at MIC levels. On the other hand, CBG demonstrated a strong inhibitory activity upon all four tested microorganisms at MICs, standing in line with previous reports of its antibiofilm activity on Gram-positive bacteria [18,19]. However, we also observed the capacity of CBG to inhibit Gram-negative biofilm formation, which has not been reported yet.

Our results indicate that CBG has a stronger antimicrobial potential than CBD. CBG and CBD have slight structural differences, namely the alicyclic ring in CBD that in CBG forms an alkyl chain [13]. This structural difference may explain the discrepancies in the results obtained since these molecules may interact differently with bacterial membrane receptors. Aqawi, Sionov, Gallily, Friedman, and Steinberg [19] demonstrated that CBG alters the membrane properties by inducing membrane hyperpolarization, decreasing the membrane fluidity while increasing its permeability. Likewise, these molecules seem to lead to a gradient disruption associated with a loss of membrane integrity [11]. Farha, El-Halfawy, Gale, MacNair, Carfrae, Zhang, Jentsch, Magolan, and Brown [18] reported that CBG exerts its bactericidal activity by acting on bacteria’s inner membrane. Moreover, the Gram-negative bacteria outer membrane hinders the uptake of both molecules, explaining the discrepancies between MICs for Gram-positive and Gram-negative bacteria tested [11]. Besides exerting an effect upon planktonic bacteria, CBD and CBG exhibited interesting results regarding biofilm formation and destruction. Reports have suggested CBG and CBD’s ability to act directly on metabolic pathways responsible for regulating biofilms, whilst suppressing metabolic activity and reducing the expression of fundamental genes [19,31]. Furthermore, CBG has been described as interfering with quorum sensing (QS) mechanisms [20], even with no detectable MIC. QS mechanisms are the basis for the development of biofilms, with these mechanisms differing extensively from Gram-positive to Gram-negative bacteria [35]. One of the pathways through which Gram-negative bacteria form biofilm is the acylated homoserine lactone (AHL) pathway [35]. There could be an interaction between this pathway and CBD and CBG, which could justify how these cannabinoids exerted biofilm inhibitory activity at sub-MIC values. On a similar note, CBD has been reported to modify the architecture of fungal biofilm through the reduction in exopolysaccharide (EPS) production and consequent thickness of the biofilm [31].

*S. epidermidis* is more abundantly found in the healthy skin microbiota, while *S. aureus* is more frequently associated with a dysbiosis state and different skin disorders [36]. Moreover, the adherence of bacteria to epithelial cells is an essential step for colonization and infection. Concerning the evaluation of cannabinoids’ impact upon bacterial adhesion to keratinocytes, it is interesting to note that both cannabinoids exerted stronger inhibition against *S. aureus* than against *S. epidermidis*. As such, the antimicrobial potential and inhibitory activity upon *S. aureus* adhesion to skin cells demonstrated by both CBD and CBG, associated with the anti-inflammatory potential as seen on the reduction in IL-1α levels and as described by several authors [5,17,37], could prove a useful alternative to ameliorate symptoms and prevent infections in patients suffering from skin disorders.

Due to the antimicrobial potential of these cannabinoids and considering a topical/dermatological application, CBD and CBG were also studied for their dermocosmetic formulations’ preservative potential. To the best of our knowledge, this is the first report concerning the use of both cannabinoids as a preservative in a dermocosmetic formulation aimed to be applied topically. CBG yielded better results than CBD as, although CBD passed the criteria for yeasts and molds, it did not lead to a reduction of ≥2 log for *P. aeruginosa*, even if it had a similar effect to CBG on the MIC/MBC assays. As cannabinoids have also been described as possessing anti-inflammatory activity, their use as multifunctional ingredients in dermatological formulations stands as a possibility. Regarding the skin microbiota, the assays performed demonstrated that both CBD and CBG formulations are microbiota-friendly, not having a significant impact on the alpha diversity of the samples. This is a good indication of the potential use of these cannabinoids in topical applications since they seem to have no significant impact on the skin microbiota of volunteers without skin diseases diagnosed. To understand in more detail their impact on the skin microbiota at a species level, qPCR was performed focusing on detecting *S. aureus* and *S. epidermidis*. Although no significant changes between donors were found for either genus or species, there seems to be a decrease in ratio regarding the relative abundance when CBD and CBG are added. Additionally, as the donors did not present skin conditions, only in 4 out of 12 donors was *S. aureus* detected, which limits the analysis of these cannabinoids’ effect on microorganisms typical of unhealthy skin. As such, the evaluation of phytocannabinoids’ impact on unhealthy skin microbiota, which demonstrates a hegemony of *S. aureus,* could be of interest in the future.

## 4. Conclusions

This report compares CBD and CBG’s antimicrobial effectiveness and further cements phytocannabinoids’ potential to be used as antimicrobial agents. Both molecules’ antimicrobial capacity strongly depends on the target microorganism, namely whether it is Gram-negative or Gram-positive. Nonetheless, we were able to determine MICs for all tested strains, including *S. pyogenes*, *E. coli*, and *P. aeruginosa.* It is of note that CBG revealed a stronger antimicrobial effect than CBD, particularly in the challenge test and in the antibiofilm assay. Further studies are needed to understand these discrepancies, as they may be connected to structural differences, receptor-binding affinity, or another mechanism other than a receptor-mediated one. Since no significant impact on the skin microbiota was observed and given its current widespread use, both CBD and CBG might be considered safe. Thus, we can assume that the development of topical formulations with active concentrations of CBG and/or CBD might represent a promising approach to tackle skin conditions where microorganisms and inflammation play a fundamental role, including psoriasis, atopic dermatitis, and acne. Nevertheless, there is still a way to go before such potential therapies can be made available in the clinical setting since doubts about these molecules’ mechanisms of action remain. A better understanding of this topic could further enhance cannabinoids’ applications and uses.

## 5. Materials and Methods

### 5.1. Cannabinoids’ Preparation

For the assays performed, cannabidiol (CBD) from three different sources and cannabigerol (CBG) from two different sources were used. A CBD isolate (CBD), purified from hemp seeds (purity ≥ 98%), was purchased from Mile High Labs (Lot: IL2004R007B; Colorado, USA. A second cannabidiol produced through chemical synthesis was purchased from Tocris Bioscience (CBD Tocris) (purity ≥ 99%; United Kingdom), while the third one, also purified from hemp seeds, was kindly provided by Linnea SA (CBD Linnea) (purity ≥ 98%, Switzerland). A CBG isolate (purity ≥ 98%), was obtained via fermentation by Amyris (Lot: 9194; Emeryville, USA), and the second CBG produced by chemical synthesis was purchased from Tocris Bioscience (CBG Tocris) (purity ≥ 99%; United Kingdom). All compounds were solubilized using ethanol (Sigma-Aldrich, Darmstadt, Germany) at 60% (*v*/*v*).

#### Cannabinoids’ Analysis by GC-MS

The profile of the cannabinoids was assessed via gas chromatography–mass spectrometry (GC-MS). To conduct this assessment, samples were dissolved in dichloromethane (DCM) (HPLC grade, 99.9%) from VWR Chemicals (Radnor, PA, USA). The derivatizing reagent N,O-bis(trimethylsilyl)trifluoroacetamide with 1% trimethylchlorosilane (BSTFA) was purchased from Merck (Darmstadt, Germany). Phytocannabinoid Mixture 10 (CRM) containing cannabidiolic acid (CBDA), cannabigerolic acid (CBGA), cannabigerol (CBG), cannabidiol (CBD), tetrahydrocannabivarin (THCV), cannabinol (CBN), tetrahydrocannabinolic acid A (THCA-A), Δ^9^-tetrahydrocannabinol (Δ^9^-THC), Δ^8^-tetrahydrocannabinol (Δ^8^-THC), and (±)-cannabichromene (CBC) was obtained from Cayman Chemical. Derivatized samples were analyzed on GC-QqQ model EVOQ (Bruker, Karlsruhe, Germany) mass spectrometer, with a Rxi-5Sil MS column (30 m × 250 μm × 0.25 μm). Helium was used as carrier gas at a constant flow rate of 1 mL/min. The conditions were as described by Attard et al. [38] with some modifications. The injector was set at 340 °C with a split of 10, and the oven temperature program was as follows: 60 °C with a hold for 1 min, then heating to 200 °C at 10 °C/min and hold for 1 min, followed by heating to 315 °C at 3 °C/min and hold for 1 min. Finally, an increase of 5 °C/min until 340 °C and hold for 15 min. The transfer line was set at 300 °C. The quadrupole was operated with an electron ionization energy of 70 eV (positive mode), source temperature at 280 °C, and a scan range of 30–1000 Da. The compound identification was made comparing the obtained mass spectra with a phytocannabinoid mixture standards, but also by comparison with the NIST library and the free online spectral repository SpectraBase^TM^ (https://spectrabase.com, accessed on 1 September 2021).

### 5.2. Determination of MIC and MLC

The minimum inhibitory concentration (MIC) was determined according to standard methods M07-A8 for aerobic bacteria, M11-A6 for anaerobic bacteria, and M27-A for yeasts [39,40,41], with some modifications. The following microorganisms were tested: five Gram-positive bacteria (*Staphylococcus aureus* DSM 799, *Staphylococcus epidermidis* LMG 10474, *Streptococcus pyogenes* DSM 20565, *Propioniferax innocua* DSM 8251, and *Cutibacterium acnes* (formerly *Propionibacterium acnes*) DSM 1897), two Gram-negative (*Escherichia coli* ATCC 25,922 and *Pseudomonas aeruginosa* DSM 1128), and one yeast (*Candida albicans* CCUG 49242). Colistin, vancomycin, and ciprofloxacin (Sigma-Aldrich, Darmstadt, Germany) were used as antimicrobial controls. These antibiotics were chosen due to their spectrum of susceptibility. Briefly, aerobic bacteria were grown overnight and inoculated in Muller Hinton Broth (MHB; Biokar Diagnostics, Beauvais, France) with cannabinoid concentrations ranging from 10 to 5000 µM. Plates were incubated on a microplate reader (Epoch, BioTek Instruments, Vermont, USA) at 37 °C during 24 h (OD: 625 nm). Two controls were simultaneously assessed: one with cannabinoids at a concentration of 5000 µM without inoculum, and other with inoculum and ethanol at 1% (*v*/*v*), which was the solvent for both CBD and CBG. The MIC was determined by observing the lowest concentration of cannabinoids where no turbidity was observed. All assays were performed in triplicate. Determination of minimum lethal concentration (MLC) was performed as described by Fernandes et al. [42]. Briefly, the MLCs were determined as the lowest concentration at which bacterial growth was prevented. This was determined by the absence of growth after inoculating aliquots of negative wells (lack of turbidity in MIC determination) on Plate Count Agar (PCA, Biokar Diagnostics, Beauvais, France). All assays were performed in quadruplicate and plated in triplicate. *Cutibacterium acnes* was grown at 37 °C in Brain Heart Infusion (BHI) broth (Biokar Diagnostics, Beauvais, France), in a Whitley A35 workstation with the anaerobic atmosphere controlled by the introduction of 10% CO_2_ and 10% H_2_ in N_2_CoA gas mix. Additionally, paraffin (Sigma-Aldrich, Darmstadt, Germany) was autoclaved, and 50 µL was added to *C. acnes* wells to ensure an anaerobic environment during the assay, which had a 48 h incubation time. *Streptococcus pyogenes* was also cultivated in BHI broth, in a microaerophilic environment using Gas Generation Sachets (Oxoid™ CampyGen™ 2.5 L Sachet; ThermoFisher Scientific, Oxford, UK) inside an Oxoid™ AnaeroJar™ 2.5 L and incubated at 37 °C for 24 h. Regarding *C. albicans*, the procedure was carried out in accordance with standard M27-A. *Candida albicans* was subcultured on Saboraud dextrose agar (SDA; Biokar Diagnostics, Beauvais, France) at 35 °C, and the inoculum was prepared fresh in saline solution just before inoculating the cannabinoids’ preparations. As with *C. acnes,* cultures were grown during 48 h for *C. albicans*. MIC and MLC determination was performed as previously stated.

### 5.3. Biofilm Formation Inhibition Assay

The biofilm formation inhibition assay was performed as described by Silva et al. [43]. *S. aureus*, *S. epidermidis*, *E. coli*, and *P. aeruginosa* were used on this assay. These were cultured in Tryptic Soy Broth (TSB; Biokar Diagnostics, Beauvais, France) at 37 °C for 24 h. Afterwards, a 96-well microplate with TSB supplemented with 1% (*w*/*v*) glucose (Sigma-Aldrich, Darmstadt, Germany) was inoculated at 2% (*v*/*v*) using an overnight inoculum (ca. 10^8^ CFU/mL) and incubated for 24 h at 37 °C with MIC and sub-MIC (½ and ¼ of the MIC) concentrations of CBD and CBG. Media without cannabinoids were used as a positive control, media without inoculum as a blank control, and ethanol (1% (*v*/*v*)) as a solvent control. Plates were processed as described by Silva, Costa, Costa, Pereira, Pereira, Soares, and Pintado [43]. All assays were performed in quadruplicate, and the results were given in biofilm formation inhibition percentage, calculated according to the following formula:

(1) $$\%\mathrm{biomass}\;\mathrm{formation}\;\mathrm{inhibition}=100-\left(\frac{OD\;assay}{OD\;positive\;control}\right)\;\times \;100$$

Minimum biofilm inhibitory concentration (MBIC) was determined as the lowest concentration at which ≥70% growth inhibition was observed in relation to the control.

### 5.4. Mature Biofilm Eradication Assay

Cannabinoids’ capacity to remove already established biofilms was assessed as described by Costa et al. [44]. CBD and CBG were used at MIC and sub-MIC concentrations (½ and ¼). Briefly, *S. aureus*, *S.* epidermidis, *E. coli*, and *P. aeruginosa* were incubated overnight in TSB at 37 °C. Bacteria were then inoculated at 2% (*v*/*v*) in TSB with 1% (*w*/*v*) glucose and incubated for 48 h at 37 °C. Afterwards, mature biofilms were exposed to MIC and sub-MIC (½ and ¼ of the MIC) concentrations of CBD and CBG. Media without compounds were used as a positive control, without inoculum as a blank control, ethanol (1% (*v*/*v*)) as solvent control, and Triton X-100 (Sigma-Aldrich, Darmstadt, Germany) at 1% (*v*/*v*) as an eradication control. Absorbance was read at 590 nm on an Epoch microplate reader (BioTek Instruments, Vermont, USA) to determine the minimum biofilm eradication concentration (MBEC). All assays were performed in quadruplicate, and MBEC was calculated using the formula below:

(2) $$\%\mathrm{biofilm}\;\mathrm{eradication}=100-\left(\frac{OD\;assay}{OD\;positive\;control}\right)\;\times \;100$$

### 5.5. Staphylococcus spp. Infection on Keratinocytes

#### 5.5.1. Keratinocytes’ Viability

HaCaT cells, an immortalized keratinocyte cell line established from adult human skin cells, were obtained from CLS Cell Lines Service (reference 300493, Eppelheim, Germany). Cells were cultured at 37 °C in a humidified atmosphere with 5% CO_2_, using Dulbecco’s Modified Eagle’s Medium (DMEM) with 4.5 g/L glucose, L-glutamine without pyruvate (Gibco, ThermoFisher Scientific, UK) containing 10% (*v*/*v*) fetal bovine serum (FBS; Gibco, ThermoFisher Scientific, UK) and 1% (*v*/*v*) penicillin–streptomycin–fungizone (Gibco, ThermoFisher Scientific, UK). Cell viability was examined with a PrestoBlue™ Cell Viability Reagent (ThermoFisher Scientific, UK) according to the manufacturer’s instructions. Briefly, cells were seeded at 1 × 10^5^ cell/mL and incubated overnight. Then, cells were washed, and the culture media were replaced with fresh culture media with different concentrations of CBD or CBG. An ethanol control was also tested at 1% (*v*/*v*), and no inhibition was verified. After 24 h of incubation, PrestoBlue™ was added, and the fluorescence was measured after 1 h of incubation, at 545 nm excitation and 590 nm emission using a Synergy HT plate reader (BioTek Instruments, Vermont, USA). Dimethyl sulfoxide (DMSO; ThermoFisher Scientific, UK) at 10% (*v*/*v*) was used as a death control, and culture media were used as a positive control for cells’ growth. All assays were performed in triplicate, with four replicas each. A cytotoxic effect is assumed for a metabolic inhibition superior to 30%, as described by ISO 10993-5 [25].

#### 5.5.2. *Staphylococcus* spp. Infection of Keratinocytes

CBG and CBD’s impact on *Staphylococcus* infection of keratinocytes was assessed. After determining which cannabinoids’ concentrations could be used, *S. aureus* and *S. epidermidis* inoculum were prepared in MHB and incubated overnight at 37 °C. Simultaneously, HaCaT were seeded at 1 × 10^6^ cells/mL in an antibiotic-free medium in 24-well plates. Plates were set up in duplicate for each strain. On the following day, the inoculum was washed thrice with phosphate buffer saline (PBS, pH 7.4) and resuspended in non-supplemented DMEM, and added to keratinocytes monolayers at a multiplicity of infection (MOI) of 20. Cocultures were maintained for 3 h at 37 °C with 5% CO_2_, after which cells were washed with PBS, trypsinized, resuspended in PBS, serially diluted, and plated in triplicate in PCA, through the drop plate method. Uninfected controls were similarly processed, with the addition of PBS instead of bacteria. In parallel, HaCaT were pre-stained with Cell Tracker Green (CMFDA; Invitrogen, ThermoFisher Scientific, Oxford, UK) before seeding, while *S. aureus* and *S. epidermidis* were pre-stained with Cell Tracker Red (CMTPX; Invitrogen, ThermoFisher Scientific, Oxford, UK) before being added to the cells. After vigorous washing, cells were imaged under a Zeiss microscope AXIO Imager.M2. Images were processed using Zen Software 3.2 (blue edition).

#### 5.5.3. Cytokines Quantification

Cytokines’ IL-1α, IL-6, and IL-8 concentrations were determined from the infected cells’ supernatants (10,000 rpm, 10 min) using an enzyme-linked immunosorbent assay (ELISA) (BioLegend, San Diego, USA), in accordance with the manufacturer’s instructions. Protein was extracted from the cells and quantified through Pierce™ BCA Protein Assay Kit (ThermoFisher, UK) and used to normalize ELISA’s results.

### 5.6. Challenge Test

To evaluate the capacity of cannabinoids to act as preservatives in cosmetic formulations, a challenge test was performed in accordance with the standard method USP 51, Antimicrobial Effectiveness Testing [26]. CBG and CBD were incorporated into a water/oil (W/O) formulation. The formulation recipe is described in Table A1. In W/O emulsion, both phases were heated at 75 °C until the ingredients melted. Both phases were slowly mixed by ultra-turrax (IKA, Germany) at 5000 rpm. The formulation was cooled until 40 °C before adding the preservative. CBG and CBD were added at a concentration of 0.5% (*w*/*w*), and the amount of water was adjusted to make the total of 100%. Phenoxyethanol (Sigma-Aldrich, Darmstadt, Germany) at 0.5% (*w*/*w*) was used as a positive control.

The formulation was divided into five containers, each challenged with one of the five method-specified microorganisms (*S. aureus* DSM 799, *E. coli* DSM 1576, *P. aeruginosa* DSM 1128, *C. albicans* DSM 1386, and *A. brasiliensis* DSM 1988) at a concentration of >1 × 10^5^ CFU/mL. All containers were made in duplicate. The volume of the suspension inoculum used was between 0.5% and 1.0% of the volume of the product, and the inoculated containers were incubated at 22.5 ± 2.5 °C. The product was evaluated at 0, 14, and 28 days. Bacteria were plated in Tryptic Soy Agar (TSA; Biokar Diagnostics, Allonne, France), while fungi were plated in Potato Dextrose Agar (PDA, Oxoid, UK). After 48 h of incubation, the viable microorganisms were counted, and the log reduction in each microorganism at each interval was reported. The effectiveness of the preservative system is determined based on the USP 51 passing criteria (Table A2).

### 5.7. Evaluation of the Impact of Cannabinoids on the Skin Microbiota

CBD and CBG’s impact on skin microbiota from healthy female volunteers (average age 30 years old) was assessed as described by Carvalho et al. [45]. All volunteers signed an informed consent form after receiving a detailed explanation about the purpose of the study. Samples were delinked and unidentified from their donors. The study was approved by the institutional review board of Universidade Católica Portuguesa approved on 17 September 2020. Female volunteers who were pregnant or during lactation period, who had performed exfoliation/skin cleansing on the face two weeks before sampling, had taken antibiotics, immunosuppressant, chronic anti-inflammatory, chronic antihistamine drugs, and/or systemic antifungals one month prior to sampling; had ingested pre- and/or probiotics two weeks before sampling; or had applied cosmetic products on the face 24 h prior to sampling were excluded from the present study. Briefly, the skin face microbiota of twelve female volunteers was collected using a cotton swab dipped in sterile PBS with 0.1% Tween 80 (ThermoFisher Scientific, Oxford, UK). Samples were incubated in RPMI (Gibco, ThermoFisher Scientific, Oxford, UK) for 16 h at 34 ± 2.5 °C with 100 rpm of agitation. In parallel, a control of the collection method was performed, consisting of a swab moistened in a sterile solution of PBS with 0.1% Tween 80 without skin microbiota, which was processed similarly to samples. Afterwards, samples were divided into four conditions: RPMI (incubation medium), cream without ingredient, cream with CBD, and cream with CBG. The formulation used was the same as the one used for the challenge test, and both CBD and CBG were at a concentration of 0.5% (*w*/*w*). All conditions were incubated overnight (approximately 18 h) at 34 °C with agitation (125 rpm). After incubation, samples were centrifuged at 15,000 rpm for 10 min, and the pellet was recovered and stored at −20 °C. DNA was extracted using PureLink™ Microbiome DNA Purification Kit (Invitrogen, ThermoFisher Scientific, Oxford, UK) in accordance with the manufacturer’s instructions and quantified using Qubit^®^ dsDNA HS (High Sensitivity) Assay Kit (Invitrogen, ThermoFisher Scientific, Oxford, UK). Analysis was performed through quantitative real-time PCR (qPCR) and next-generation sequencing to evaluate changes in microbial populations.

#### 5.7.1. 16S rRNA Gene and ITS2 Region Amplification and Sequencing

The V3-V4 hypervariable regions of the 16S rRNA gene were amplified using universal primers fused with Illumina adapters sequences 16S_F_ngs 5′-TCGTCGGCAGCGTCAGATGTGTATAAGAGACAGCCTACGGGNGGCWGCAG-3′ and 16S_R_ngs 5′-GTCTCGTGGGCTCGGAGATGTGTATAAGAGACAGGACTACHVGGGTATCTAATCC-3′ (Integrated DNA Technologies). The ITS2 region was amplified using ITS2_F_ngs 5′-TCGTCGGCAGCGTCAGATGTGTATAAGAGACAGGTGARTCATCRARTYTTTG-3′ and ITS2_R_ngs 5′-GTCTCGTGGGCTCGGAGATGTGTATAAGAGACAGTCCTSCGCTTATTGATATGC-3′. The PCR reactions were performed in 25 µL 1 × AmpliTaq Gold 360 Master Mix (Applied Biosystems, Foster City, CA, USA), 0.2 µM to 0.4 µM of forward and reverse. Microbial DNA-free water (Qiagen, Germany) was added to PCR-negative controls instead of DNA. Amplicons underwent a purification step with magnetic beads using the Axy Prep PCR Clean-Up Kit (Axygen, Union City, CA, USA) and visualized in 1.5% agarose gels. Their concentration was determined with the Qubit dsDNA HS Assay Kit (Life Technologies, Foster City, CA, USA). Equal amounts of amplicons were used for sequencing library construction using the Illumina 16S Metagenomic Sequencing Library preparation protocol. The final sequencing library was sequenced with MiSeq Reagent Kit v3 (Illumina, San Diego, CA, USA) in the Illumina MiSeq platform, using 300 bp paired-end sequencing reads with an expected output of 100,000 reads per sample.

#### 5.7.2. Sequencing Data Analysis

The analysis of the generated raw sequence data was carried out using QIIME2 v2021.4 [46]. The reads were denoised using the DADA2 plugin, which included trimming and truncating low-quality regions, dereplicating the reads, and filtering chimeras [47]. The filtered reads were organized into operational taxonomic units (OTUs) and then classified by taxon using the SILVA (release 138 QIIME) database, with a clustering threshold of 99% similarity. Only OTUs containing at least ten sequence reads were considered significant.

#### 5.7.3. Determination of Relative Abundance of *Staphylococcus* Genus, and *S. aureus* and *S. epidermidis* Species

Real-time quantitative polymerase chain reaction (qPCR) assays were used to quantify the relative abundances of *Staphylococcus* genus and of *S. aureus* and *S. epidermidis* species. For that, a universal assay composed of universal primers targeting a conserved region of the 16S rRNA gene; and a genus- or specie-specific assay, composed of primers targeting genus- or specie-specific genes, were used [48,49,50,51]. Primers used to determine *Staphylococcus* abundance by qPCR are shown in Table A3. qPCR reactions were prepared to a final volume of 10 μL, containing 1 × NZYSupreme qPCR Green Master Mix (NZYtech. Lisbon, Portugal), 0.5 to 1 μM of forward and reverse primers (Integrated DNA Technologies, IDT, Heverlee, Belgium), 2 μL of microbial DNA-free water (Qiagen, Hilden, Germany) and 1 μL of DNA. The qPCR was performed in a qTOWER^3^ G (Analytik-Jena,Hilden, Germany) with the following conditions: 10 min at 95 °C, followed by 40 cycles of denaturation at 95 °C for 15 s and annealing/extension at 60 °C for 1 min. The amplification steps were followed by a melt dissociation step to check for nonspecific product formation. In addition, the PCR product purity was also controlled by 2% agarose gel electrophoresis. Two replicates were performed for each sample. To exclude any potential environmental contaminant in qPCR reactions, blanks were prepared using microbial DNA-free water (Qiagen, Hilden, Germany) instead of DNA. Positive controls for each of the bacterial assays were included. The relative standard curve method was used to quantify the total microbial load and the specific microbial genera or species. To create standard curves, dilution series of known microbial CFU number were used to create a standard curve for each pair of primers, by plotting the Log_10_ of each known CFU number in the dilution series against the determined threshold cycle (Ct) value. For each genus and species, the relative abundance was calculated using the Log10 ratio between the CFU number determined for the genus- or specie-specific assay and the CFU number determined for the universal assay. To reduce the inter-individuality, for each volunteer, a ratio between the condition test and its control condition was calculated.

### 5.8. Statistical Analysis

Statistical analysis was performed using IBM SPSS Statistics v21.0.0 (New York, NY, USA) software. Normality of the distributions was evaluated using Shapiro–Wilk’s test. For the data which followed a normal distribution and where assumption of homoscedasticity was met, one-way analysis of variance (ANOVA) coupled with Turkey’s post hoc test was used to assess the differences between the results observed, with differences being considered significant for *p* values below 0.05. When the data did not exhibit a normal distribution, a mean comparison between independent samples was carried out using Mann–Whitney’s test. Additionally, a paired-samples t-test was performed for the biofilms’ assays, with differences being considered significant for *p* values below 0.05. For the skin microbiota study, GraphPad Prism 6 software was used, and a one-way ANOVA followed by Holm–Sidak’s multiple comparisons test was performed to compare the alpha diversity of cream without ingredient with cream with CBG and cream with CBD, with differences being considered significant for *p* values below 0.05.

## Figures and Tables

**Table 1:** Results for MIC and MLC assays using cannabinoids and different antibiotics as control. Data from 4 biologically independent samples for each isolate. Concentrations are expressed in µM.

Compound	*S. aureus*		*S. epidermidis*		*S. pyogenes*		*C. acnes*		*P. innocua*		*P. aeruginosa*		*E. coli*		*C. albicans*	
	MIC	MBC	MIC	MBC	MIC	MBC	MIC	MBC	MIC	MBC	MIC	MBC	MIC	MBC	MIC	MBC
CBG	25	75	25	50	50	75	500	3180	10	50	400	5000	500	5000	200	400
CBG Tocris	10	25	25	75	75	100	1000	3180	10	25	400	3180	1000	3180	400	500
CBD	10	75	10	25	25	50	500	5000	25	75	750	5000	750	5000	200	400
CBD Tocris	75	100	50	75	50	100	>1000	>5000	75	100	1000	3180	3180	3180	250	500
CBD Linnea	10	25	5	10	10	25	300	5000	10	50	1000	>5000	3180	>5000	200	300
Vancomycin	0.34	0.7	0.17	0.34	0.17	0.34	0.7	1.4	0.09	0.17	-	-	-	-	-	-
Ciprofloxacin	3	6	0.3	0.75	1.5	3	0.3	0.75	0.3	0.3	3	6	6	15	-	-
Colistin	-	-	-	-	-	-	-	-	-	-	0.4	4.3	0.4	0.9	-	-

**Table 2:** Log variation registered at 14 days for the microorganisms tested. No increase is defined as no more than 0.5 log units higher than the previous value measured.

Log Variation	Day 14				Day 28			
	CBG	CBD	No Preservative	Phenoxyethanol	CBG	CBD	No Preservative	Phenoxyethanol
*Staphylococcus aureus*	2.1	2.0	0.7	6.0	No increase			
*Escherichia coli*	2.0	2.0	0.4	2.1				
*Pseudomonas aeruginosa*	2.0	0.8	−0.5	1.5				
*Candida albicans*	No increase							
*Aspergillus brasiliensis*	No increase		Increase	No increase	No increase		Increase	No increase

## Data Availability

The data presented in this study will be openly available and are currently under submission.

## Appendix A

**Table A1:** Composition of the different phases used in the production of water/oil emulsion.

Vendor	Phase I	(*w*/*v*) %
AAK (Malmö, Sweden)	Akoline PGPR	5.00
Aprinnova (USA)	Neossance Squalene	5.00
Acofarma (Madrid, Spain)	Caprylic/Capric triglyceride	7.00
	Vaseline	10.00
	Lanoline	10.00
	Beeswax	1.80
	Magnesium Stearate	1.00
	Phase II	
	Deionized water	55.95
Acofarma (Madrid, Spain)	Glycerin	3.00
	Sodium Chloride	0.75
	Phase III	
	Preservative	0.50
	Total	100.00

**Table A2:** The criteria for category 2 products (topically used products made with aqueous bases or vehicles, nonsterile nasal products, and emulsions, including those applied to mucous membranes) (The United States Pharmacopeial Convention (2011) USP 51).

Bacteria	Not less than 2.0 log reduction from the initial count at 14 days, and no increase from the 14 days count at 28 days
Yeast and Moulds	No increase (not more than 0.5 log unit higher than the previous value measured) from the initial calculated count at 14 and 28 days.

**Table A3:** Primers used for the determination of *Staphylococcus* abundance by qPCR.

Primer	Forward Primer (5′ ->3′)	Reverse Primer (5′->3′)	Reference
Universal Bacteria	TCCTACGGGAGGCAGCAGT	CGTATTACCGCGGCTGCTGGCAC	[48]
*Staphylococcus*	GGCCGTGTTGAACGTGGTCAAATCA	YATHACCATTTCWGTACCTTCTGGTAA	[49]
*S. aureus*	AGGACAATCATGGCAAGCGTAC	AACGGACAACATCTAAACTGGC	[50]
*S. epidermidis*	GGCAAATTTGTGGGTCAAGA	TGGCTAATGGTTTGTCACCA	[51]

**Table A4:** Purity of the CBD and CBG samples.

	Purity (%)	SD
CBD Amyris	98.63	0.37
CBD Tocris	98.44	1.02
CBD Linnea	99.36	0.40
CBG Amyris	99.35	0.02
CBG Tocris	99.48	0.19
